# Building health literacy system capacity: a framework for health literate systems

**DOI:** 10.1093/heapro/daab153

**Published:** 2021-12-12

**Authors:** Kristine Sørensen, Diane Levin-Zamir, Tuyen V Duong, Orkan Okan, Virginia Visconde Brasil, Don Nutbeam

**Affiliations:** 1 Global Health Literacy Academy, Risskov, Denmark; 2 Department of Health Education and Promotion, Clalit, Tel Aviv and School of Public Health, University of Haifa, Haifa, Israel; 3 School of Nutrition and Health Sciences, Taipei Medical University, Taipei, Taiwan; 4 Interdisciplinary Centre for Health Literacy Research, Bielefeld University, Germany; 5 School of Nursing, Universidade Federal de Goiás, Goiânia, Brazil; 6 Sydney School of Public Health, University of Sydney, Sydney, Australia

**Keywords:** health literacy, capacity building, health literate system, health literate organization, system performance indicator

## Abstract

The human and social implications of poor health literacy are substantial and wide-ranging. Health literacy represents the personal competencies and organizational structures, resources and commitment that enable people to access, understand, appraise and use information and services in ways that promote and maintain good health. A large-scale societal improvement of health literacy will require political buy-in and a systematic approach to the development of health literacy capacity at all levels. This article builds the case for enhancing health literacy system capacity and presents a framework with eight action areas to accommodate the structural transformation needed at micro, meso and macro levels, including a health literate workforce, health literate organization, health literacy data governance, people-centred services and environments based on user engagement, health literacy leadership, health literacy investments and financial resources, health literacy-informed technology and innovation, and partnerships and inter-sectoral collaboration. Investment in the health literacy system capacity ensures an imperative and systemic effort and transformation which can be multiplied and sustained over time and is resilient towards external trends and events, rather than relying on organizational and individual behavioural change alone. Nevertheless, challenges still remain, e.g. to specify the economic benefits more in detail, develop and integrate data governance systems and go beyond healthcare to engage in health literacy system capacity within a wider societal context.

## Lay Summary

Health literacy represents the personal competencies and organizational structures and resources enabling people to access, understand, appraise and use information and services in ways that promote and maintain good health. To meet the needs related to impact of poor health literacy, this article introduces a framework for the development of health literacy system capacity with eight action areas including the development of a health literate workforce, health literate organization, health literacy data governance, people-centred services and environments based on user engagement, health literacy leadership, health literacy investments and financial resources, health literacy-informed technology and innovation, and partnerships and inter-sectoral collaboration. Investment in health literacy system capacity ensures a future-proof effort that can be multiplied and sustained over time, rather than relying on organizational or individual behavioural change alone.

## INTRODUCTION

The human and social implications of poor health literacy are substantial and wide-ranging (Berkman *et al.*, 2011). As a modifiable determinant of health (Nutbeam and Lloyd, 2021), health literacy represents the personal knowledge and competencies which accumulate through daily activities, social interactions and across generations. Personal knowledge and competencies are mediated by the organizational structures and availability of resources which enable people to access, understand, appraise and use information and services in ways that promote and maintain good health and wellbeing for themselves and those around them (Nutbeam and Muscat, 2021). Thus, the way that organizations and societal systems are supporting health literacy becomes an important factor for the development of healthier populations.

However, surveys around the globe reveal that large proportion of populations are classified with poor health literacy, although, significant differences exist between countries (Rudd, 2007; Sørensen *et al.*, 2015; Duong *et al.*, 2020). Health literacy is independently linked to increased healthcare utilization and costs (Haun *et al.*, 2015), e.g. higher rates of emergency service use, prolonged recovery and complications (Betz *et al.*, 2008; Palumbo, 2017); as well as poorer management of chronic disease and less effective use of medications (Persell *et al.*, 2020). People with poor health literacy are also less responsive to traditional health education and make less use of preventive health services, such as immunization and health screening (Kino and Kawachi, 2020). This feature has been especially relevant during the COVID-19 pandemic (Paakkari and Okan, 2020). Limited research examining the economic consequences of poor health literacy indicates significant additional costs for healthcare services ranging from the US $143 to $7.798 annually and, additional costs on a system level of expenditure ranging from 3% to 5% of total healthcare costs (Eichler *et al.*, 2009). Meeting the needs of people with limited health literacy could potentially save appropriately 8% of total costs (Haun *et al.*, 2015).

The social distribution of poor health literacy compounds the impact of wider social and economic determinants of health (Nutbeam and Lloyd, 2021). While it cannot be seen as a panacea, health literacy is one of the few social determinants of health that responds to individual/behavioural interventions to increase personal capabilities and be mitigated by reducing the situational demands experienced by people in different settings, e.g. hospital, clinic, community (Nutbeam and Muscat, 2020; Nutbeam and Lloyd, 2021). Besides, health literacy has a strong ethical underpinning as a means to achieve health equity at individual level (Nairn, 2014; Levin-Zamir and Bertschi, 2018; Paakkari and George, 2018) and societal level (Stormacq *et al.*, 2019).

Large-scale societal improvement of health literacy requires political buy-in and a systemic approach to develop health literacy capacity at all levels (Trezona *et al.*, 2018). It is, therefore, critical that governments and health providers among others acknowledge the call to action and recognize their role in the improvement of health literacy from a structural point of view (Koh *et al.*, 2012). To facilitate and support such important changes, several countries have already adopted national health literacy policies or incorporated health literacy as priority in wider health strategies and policy frameworks. Most of those have their primary focus on advancing health literacy to improve clinical quality and safety, health services efficiency, and deliver better outcomes for patients as these approaches carry strong political weight (Australian Commission on Safety and Quality in Health Care, 2014; Scottish Government, 2018). By contrast, a broader focus on improving health literacy beyond the health sector is less common and less implemented despite the great potential for advancing health literacy across the life course (Maindal and Aagaard-Hansen, 2020).

System challenges do not affect a single component nor a single sub-system. Health literacy as a systemic challenge is deeply rooted. The problems featured by low health literacy are re-produced despite attempts to fix them from within the system and in turn, creating a persistent pattern of system failure. A proper response will require coordination across many governmental departments and agencies, as well as the private sector and civil society. This article, therefore, seeks to build the case for strengthening health literacy system capacity and propose a framework accommodating the structural support needed to overcome the detrimental impact of low health literacy.

## BUILDING THE CASE FOR HEALTH LITERACY SYSTEM CAPACITY

Strengthening health systems’ capacity requires a combination and integration of actions which can be accommodated by integrating health literacy as a key issue in public health policies, as well as in educational, social welfare policies at the local, national and international level (Van den Broucke, 2019). Building the case for health literacy system capacity, some challenges and opportunities should be emphasized.

### Health literacy is a political choice

Across the world, systemic health literacy policies and strategic planning have emerged as governments become aware of its importance for population health (Rowlands *et al.*, 2018; Trezona *et al.*, 2018). Austria has adopted health literacy as 1 of 10 national health goals (Bundesministerium für Gesundheit, 2013). The USA launched a national action plan on health literacy in 2010 informed by town hall meetings with relevant stakeholders (U.S. Department of Health and Human Services, 2010). As a reaction to health literacy population research Germany released a national action plan to make the health system more user-friendly and establish health literacy as a standard on all levels of the health system (Schaeffer *et al.*, 2018, 2020). Moreover, the Portuguese health literacy action plan aimed to empower people across the life course targeting children, the elderly, and people in the working-age population, who are in the prime of their lives, to lower levels of mortality and morbidity (Saúde, 2018). Scotland focused on making it easier for the public to access and understand health services (NHS Scotland, 2014; Scottish Government, 2018). Importantly, these sorts of health literacy strategies bridge preventable health inequities through inclusion by ensuring timely and meaningful access to services, programmes and activities based on health literacy, language access and cultural competency as integral parts of the delivery of safe, quality, people-centred care and public services (Rowlands *et al.*, 2017).

*However, while a political mandate requires an adequate systemic response and capacity, thus far the policies and strategies often lack the corresponding systemic response and capacity building to conduct and carry out the actions described.*

### Organizational health literacy is necessary, but not sufficient for systemic transformation

Public health and social service organizations are responsible for promoting health literacy and provide equitably accessed services and information (Trezona *et al.*, 2017). The concept of organizational health literacy refers to the degree to which organizations equitably enable individuals to find, understand, and use information and services to inform health-related decisions and actions for themselves and others (Centers of Disease Control and Prevention, 2020). It is getting traction along with the concept of health literacy responsiveness which addresses the provision of services, programs and information in ways that promote equitable access and engagement, that meet the diverse health literacy needs and preferences of individuals, families and communities, and that support people to participate in decisions regarding their health and social wellbeing (Trezona *et al.*, 2017). The 10 attributes of health literate organizations provide a much-cited framework for organizational development to enhance health literacy (Brach *et al.*, 2012). In addition, the organizational health literacy is recommended as a quality improvement measure which can be immediately applied (Brega *et al.*, 2019).

*Yet, while organizational health literacy is necessary to create the transition towards health literate organizations; it is not, however, sufficient to fully embrace the transformation needed to build health literate systems with a sustainable impact.*

### Health literacy is a critical source of empowerment

Health literacy is an asset and a critical source of empowerment (Kickbusch, 2008) that leads to higher levels of critical consciousness, including questioning and reflecting; a sense of power, self-esteem and self-efficacy; and an understanding of how to make use of all available resources to engage in social and political actions (Crondahl and Eklund Karlsson, 2016). Empowerment refers to a social action process for people to gain mastery over their lives in the context of changing their social and political environment to improve equity and quality of life (Wallerstein *et al.*, 2015). Empowerment of the public and patients, as well as health workers, is required with the healthcare information they need to recognize and assume their rights and responsibilities to access, use and provide appropriate services and to promote health and prevent, diagnose and manage disease (World Medical Association, 2019).

*Health literacy systems can act as catalyst for health literacy as an asset and critical source of empowerment. However, presently, health literacy remains an untapped and under-developed human resource in many parts of the world.*

### Health literacy reduces disparities as a modifiable determinant of health

Health literacy helps reducing the disparities regarding ethnicity (Paasche-Orlow and Wolf, 2010). People’s social and cultural contexts are inextricably linked to how they perceive and act on health information and how they derive meaning. If cultural norms do not match up with the dominant values of a certain health system, even people with adequate health literacy may have trouble accessing health services, communicating with providers and pursuing effective self-management. Such cultural mismatches—along with low socioeconomic levels and historic discrimination—have contributed to disparities in health and healthcare experienced by individuals in racial, ethnic and linguistic minority groups. An adequate response is needed, e.g. by use of cultural competency which refers to the ‘practices and behaviours that ensure that all patients receive high-quality, effective care irrespective of cultural background, language proficiency, socioeconomic status, and other factors that may be informed by a patient’s characteristics' (OMH, 2019). Improving the socioeconomic and cultural appropriateness of health materials, personal interactions and services is an important step towards addressing low health literacy among diverse populations. Increased efforts to improve cultural competency and health literacy of health professionals and the systems in which they practice can improve consumer and patient satisfaction, health outcomes and reduce the cost of care and disparities (McCann *et al.*, 2013).

*Notably, across the world, disparities related to gender, age, race/ethnicity need to be dealt with in a structural manner in which, health literacy can play a significant role.*

These lessons learned emphasize the role of health literacy in developing fit-for-purpose systems and making use of untapped human resources while also protecting people in vulnerable situations through strong commitment, governance and political leadership.

## A FRAMEWORK FOR BUILDING HEALTH LITERACY SYSTEM’S CAPACITY

**Fig. 1:**
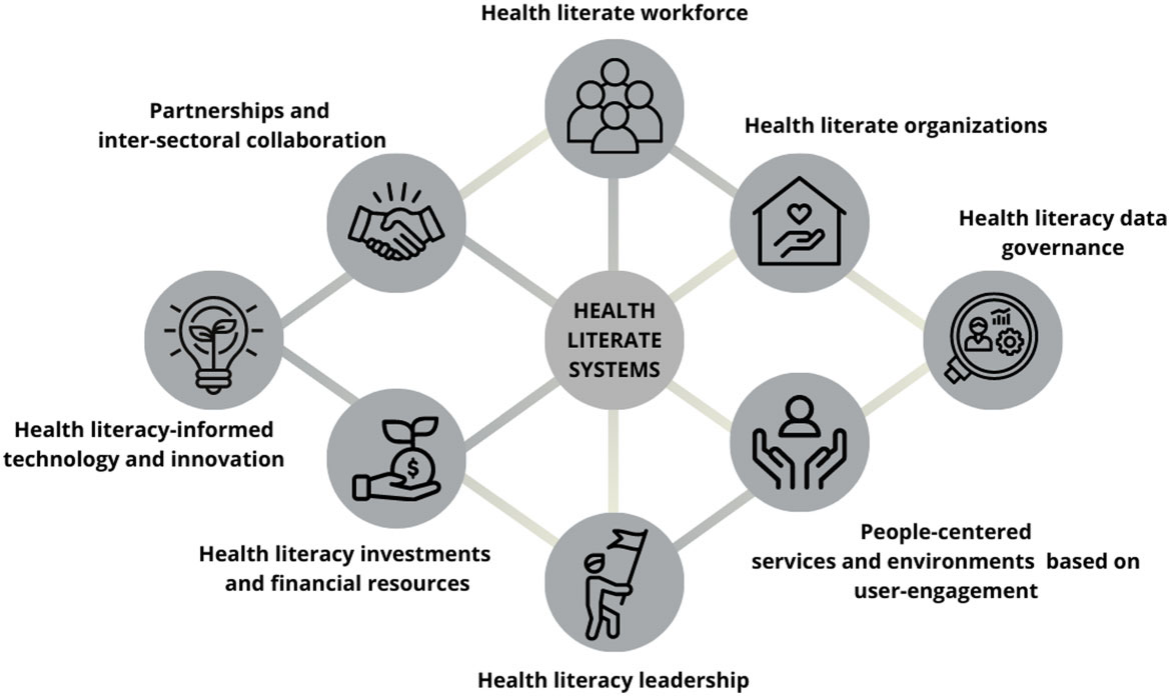
Building health literacy system capacity: a framework for health literate systems.

Inspired by previous models of public health capacity systems (Aluttis *et al.*, 2013; Pederson *et al*., 2017, p.11), an adapted health literacy system capacity framework (Figure 1) is proposed which addresses systemic capacities such as the workforce, organizational structures, research and knowledge development, financial resources, partnerships, leadership and good governance, technology and innovation as well as people-centredness based on user engagement and enabling environments. Capacity building related to public health entails the development of sustainable skills, organizational structures, resources and commitment to prolong and multiply health gains many times over (Hawe *et al.*, 1997). Capacity building ensures that the conditions are in place to achieve health improvement and that systemic effort can be multiplied and sustained over time, independent of external events (Aluttis *et al.*, 2013).

### Health literate workforce

While studies regarding the health literacy of professionals are at an early stage (Cafiero, 2013), it is widely accepted that they play a pivotal role in helping people understand their health and healthcare. Improving the sensitivity and responsiveness of clinicians and health service management to the impact of low health literacy helps to minimize disadvantages and health outcomes (Wittenberg *et al.* 2018). Attempts have been made to develop a list of competencies related to health literacy (Coleman *et al.*, 2013; Karuranga *et al.*, 2017). Capacity building related to the workforce, representing a wide range of disciplines and sectors include the human resources and their competencies; training and development; and professional associations. Increasing health professionals’ health literacy skills and facilitating their use of health literacy strategies has the potential to change clinical and community practice and support improved health outcomes (Cafiero, 2013). Correspondingly, in recent years, health literacy has become an emerging professional skill in job adverts for a wide range of professions (Sørensen, 2014) and organizations (Arnold and Wade, 2015). Global collaboration on professional development is fostered through, for instance, the International Health Literacy Association (www.i-hla.org) and the International Union of Health Promotion and Education (www.iuhpe.org). However, further work is needed to develop educational curricula and guidelines on health literacy for professional developments of various disciplines within the health sector, educational sector and beyond.

### Health literate organizations

Capacity building for health literacy in organizations relates to their structure and entails the institutional capacity, the programme and service delivery structures and emergency response system. According to Brach *et al.* (2012), organizational health literacy capacity can be based on ten attributes: leadership, integration of health literacy in planning and evaluation, a health literate workforce, user engagement in design and implementation, avoiding stigma, health literate communication skills and strategies, easy access to information and services, easy understandable and enabling designs, meeting the needs of users in high-risk situations, and transparency about costs and coverages (Brach *et al.*, 2012). In recent years, numerous other toolkits and resources have been developed to guide the efforts of improving organizational health literacy (Dietscher and Pelikan, 2017; Farmanova *et al.*, 2018; Trezona *et al.*, 2018).

### Health literacy data governance

Health surveillance is an essential part of the operation of health systems today (Rechel *et al.*, 2019). While, health literacy assessment research is growing across the world (Okan *et al.*, 2019), the implementation of systemic health literacy data and monitoring mechanisms is scarce. Health literacy analytics is important to inform the development of health literacy policy and practice. Data—combined with analytics—are a uniquely valuable asset for any societal system to strengthen management, operational optimization, user insights, personalization and forecasting (McKinsey, 2021). Health literacy data and analytics can be applied to design an effective data strategy and understand the core value of the data; ensure returns for health literacy data investments; identify the right architecture, technology decisions, and investments required to ensure the ability to support future health literacy data capabilities; apply proper governance in an agile way to ensure the right balance of data access and data security; create a data organization and culture that harness ethics and security, and train the workforce to use the health literacy data as a tool in everyday decision-making. The development and integration of health literacy as a key performance indicator and health literacy data governance including data availability, usability, consistency, integrity and security as part of health literacy systems’ capacity remains an important challenge to accomplish as part of health literacy systems’ capacity.

### People-centred services based on user engagement and enabling environments

Health systems oriented around the needs of people and communities are more effective, cost less, improve health literacy and patient engagement, and are better prepared to respond to health crises (World Health Organization, 2017). According to WHO, integrated people-centred health services place people and communities, not diseases, at the centre of health systems, and empower people to take charge of their own health rather than being passive recipients of services (2017). Health literacy capacity is a foundation for people-centred services and environments (Messer, 2019) and has been recognized as a key component of performance metrics to ensure the collaborative model of patient-centred care (Yunyongying *et al.*, 2019), in particular with regards to patient activation (i.e. the knowledge, skills and confidence to become actively engaged in their healthcare) which increases health outcomes (Yadav *et al.*, 2019). Moreover, patient-centred communication has been found to be a valid model to improve health literacy and patient satisfaction (Altin and Stock, 2015) and patient outcomes (Gaglio, 2016). Health literacy informs patients and providers to co-produce health and engage in shared decision-making by focusing on the communication and deliberation process during a healthcare encounter (Henselmans *et al.*, 2015; McCaffery *et al.*, 2016) and social mobilization in communities (Levin-Zamir *et al.*, 2017).

Co-creation is a foundation for enabling and empowering stakeholders as part of system’s health literacy responsiveness. Previous frameworks on organizational health literacy have highlighted the importance of user engagement through (Brach *et al.*, 2012; International Working Group Health Promoting Hospitals and Health Literate Healthcare Organizations, 2019). The involvement of all relevant stakeholders in the design and evaluation of documents, materials and services helps to ensure that their development and implementation are adequate in addressing the needs of these stakeholders (Thomacos and Zazryn 2013). Adhering to user experience and health literacy principles when developing consumer health information systems can improve a user’s experience as well as reducing implications on patient safety (Monkman and Griffith, 2021).

Building health literate systems include the establishment of creating enabling environments. Health literacy is closely related to the strategy of *making healthy choices easy choices—*an expression coined by Nancy Milio. Milio (1987) challenged the notation that a main determinant for unhealthful behavioural choice is lack of knowledge and highlighted that governmental and institutional policies set the range of options for personal choice making. Importantly, the political commitment of creating enabling environments that support health literacy and healthy choices through pricing policies, transparent information and clear labelling is exemplified in the *Shanghai declaration on promoting health the 2030 agenda for sustainable development goals* (World Health Organization, 2016).

### Partnerships and inter-sectoral collaboration

The health literacy systems’ capacity can be increased through the creation of formal and informal partnerships; joint ventures; and public-private partnerships. A wide range of health literacy partnerships and collaborations are already present in the form of interest groups, networks and platforms within the health literacy field (Sørensen *et al.*, 2018). Through patience, perseverance and continuous open communication and learning, ripple effects can be created into local and national policy and practice by the collective efforts of the key stakeholders involved (Estacio *et al.*, 2017). Health literacy partnerships involving public and private stakeholders, communities and the civic society can enhance the impact of health literacy to ensure that the most disadvantaged population groups are reached (Dodson *et al.*, 2015). Partnerships and inter-sectoral collaboration can furthermore accelerate the work on health literacy as a social determinant of health by influencing root causes related to health and wellbeing which are often to be found outside the health sector (Nutbeam and Lloyd, 2021).

### Health literacy-informed technology and innovations

As a result of the exponential technical revolution, health literacy-informed technology and innovations become a way to influence digital and technical developments for the public good. Digital health literacy, for instance, is an important tool for equitable access to digital resources (Levin-Zamir and Bertschi, 2018) and for supporting communities (Walden *et al.*, 2020). Originally, the concept focused on electronic sources of health information (Norman and Skinner, 2006), yet, with increased opportunities for promoting health through interactive virtual tools, digital health literacy now also reflects, a more sophisticated understanding of using these resources (van der Vaart and Drossaert, 2017). However, health literacy disparities exist in the use of technologies for health management (Meyers *et al.*, 2019). In the digital era, it is critical not just to provide the information but also support tools to help receivers seek, evaluate and analyse the quality of information that are important to improve health literacy and health (Karnoe *et al.*, 2018; Keselman *et al.*, 2019). Building the health literacy capacity at systems’ levels, therefore, need to embrace and integrate digital health and social innovations, social media platforms and digital resources which are critical to the advancement of individual and population health (Levin-Zamir and Bertschi, 2018).

### Health literacy investments and financial resources

A health systems’ capacity concerning investments and financial resources refers to the generation of financial resources and resource allocation, e.g. through tax and treasury, insurance and donations. Health systems vary within and between countries which means that financial resources are distributed differently. The cost-effectiveness of health literacy and its social and economic return on investment is a growing area of interest based on the added value of interventions, campaigns and programmes. For instance, in a recent health literacy review within the European region of the World Health Organization, the economic return ratios ranged from 0.62 to 27.4 and social return on investments varied between 4.41 and 7.25 (Stielke *et al.*, 2019). It is previously estimated that low health literacy may accommodate for 3–5% of total healthcare costs in the USA (Eichler *et al.*, 2009) because health literacy is inversely associated with healthcare utilization and expenditure. A study by Vandenbosch *et al.* (2016) showed, for instance, that low health literacy was associated with more admissions to 1-day clinics, general practitioner home consultations, psychiatrist consultations and ambulance transports as well as with longer stays in general hospitals. Strategic investments and allocation of financial resources to health literacy advancement are, therefore, necessary elements of building health literacy system capacity.

### Health literacy leadership

*The Shanghai Declaration on Health Promotion* highlights health literacy and good governance as a priority for modern health systems (World Health Organization, 2016). The Commission on Safety and Quality in Healthcare in Australia, for instance, leads by example in the way they raise awareness about health literacy management by encouraging leaders to put systems in place that ensure education and training for the workforce, emphasize whole-of-organization policies which embed health literacy considerations into existing processes; accommodate use of easily understood language and symbols; and embrace consumers by having processes in place to provide support for consumers with additional needs (Australian Commission on Safety and Quality in Health Care, 2014). Notably, health literacy champions are in demand as change agents to facilitate the systemic and organizational implementation of health literacy (Sørensen, 2021). Health literacy leadership can be characterized by management-buy-in, pioneer spirit, endurance, persistence and confidence that health literacy is a public good. It is encouraged to include health literacy management skills as part of education and professional development (Sørensen, 2021). Several leadership projects and initiatives have been developed in research and practice to improve health literacy and outcomes in relation to social work, nursing and preventing non-communicable diseases (Liechty, 2011; Stikes *et al.*, 2015; Manhanzva *et al.*, 2017).

## BUILDING SYSTEMS’ HEALTH LITERACY—A VIABLE WAY FORWARD

Building the case for health literacy, it becomes clear that the way health literacy is being approached today—with a primary focus on individual and organizational change—is necessary but not sufficient. Health literacy challenges are *complex* (Stroh, 2015) and often characterized by a *structural mismatch between engaged institutions, the context they work in and the needs they facilitate* (Leadbeater and Winhall, 2020). In order to build better systems that are fit for purpose; it is imperative to recognize the untapped potential of health literacy as an asset to overcome system failure and present systems’ health literacy capacity as an innovative solution for a viable way forward. Only when systemic opportunities and new innovations appear, the health literate system gains momentum and become effective. Importantly, health literacy framed as a strategic approach to systemic solutions is emerging in political debates related to health system reforms, patient empowerment and shared decision-making (Weishaar *et al.*, 2019). For the first time, for instance, the US Department of Health and Human Services integrates health literacy as part of its framework to achieve the Healthy People 2030’s goals (Brach and Harris, 2021).

According to Leadbeater and Winhall (2020), the act of transforming systems will only come about if changes are happening in combination at the micro, meso and the macro levels of the system. Thus, with regards to health literacy the systems’ capacity can be induced by radical new solutions, habits and ways of living at micro level among people and professionals involved; in combination with broader changes at macro level in the form of ‘new landscapes’ formed by changes in societal values and political ideologies, demographic trends and economic patterns which shape the context in which the system operates. Yet, changes at micro and macro levels are not enough; there needs to be a change at meso level too where previous ‘regimes’ being the combination of institutions, technologies, markets and organizations that give a system its structure, give way for new ones to emerge. Hence, by upskilling the system’s health literacy capacity at meso level; the system will advance its ability to respond adequately to the daily needs of people served as well as to the contextual demands such as ageing populations, digitization and people-centred care.

Dislodging incumbent systems is often a challenge in itself as ‘systems are often hard to change because power, relationships and resources are locked together in a reinforcing pattern according to the current purpose. Systems start to change when this pattern is disrupted and opened up and a new configuration can emerge’ especially regarding power, relations and flow of resources (Leadbeater and Winhall, 2020, p. 31). Currently, there are cracks in the way health ecosystems are primarily based on the purpose of delivering healthcare. Health literacy as a modifiable determinant of health is likely to expand the cracks by disrupting the status quo as it changes the power of the patients/users; the relations between patients and professionals, and the flow of resources based on, e.g. priorities related to people-centred care and value-based distribution of services.

To follow international progress, it is paramount to acknowledge health literacy as a system performance indicator and develop a data governance system which can generate, analyse and integrate the health literacy data into international monitoring mechanisms. The systemic approach for population data collection is characterized by work in progress. The European Health Information Initiative (EHII) of the WHO—which fosters international cooperation to support the exchange of expertise, build capacity and harmonize processes in data collection and report acts—as catalyst in this regard (https://www.euro.who.int/en/data-and-evidence/european-health-information-initiative-ehii. 27 September 2021, last date accessed). EHII hosts the Action Network of the Measurement of Population and Organizational Health Literacy consisting of partners from 28 Member States in Europe and beyond to enable evidence-formed policy and practice ( https://m-pohl.net/. 27 September 2021, last date accessed). Upscaling from a European to a global context is still needed. However, previous population studies in Asia seem promising (Duong *et al.*, 2017) and can help pave the way for the development of a global population health literacy indicator.

The article outlined the detrimental impacts of poor health literacy, built the case for health literacy system capacity and presented a framework on how to develop health literate systems. Essentially, the health literacy system capacity framework aims to facilitate a systemic transformation which can be multiplied and sustained over time, and which is resilient towards external trends and events, rather than relying on individual behavioural change or organizational change alone to overcome the challenge of poor health literacy. Furthermore, an enhanced health literacy system capacity prevents system failure by ensuring a better match between the organizations, the context they work in and the needs they meet by addressing and enhancing the capacity of the workforce, organizational structures, data governance, financial resources, partnerships, leadership, technology and innovation as well as people-centred services and environments based on user engagement. Nevertheless, challenges remain, e.g. to specify the economic benefits more in detail; develop and integrate data governance systems and go beyond healthcare to engage in health literacy system capacity within a wider societal context.

## CONFLICT OF INTEREST

The authors have nothing to declare.

## DISCLAIMER

The authors alone are responsible for the views expressed in this article and they do not necessarily represent the views, decisions or policies of the institutions with which they are affiliated.
